# Some "Bloated Aristocrats" of Physic

**Published:** 1892-07-09

**Authors:** 


### The Doctor's Armchair.

SOME "BLOATED ARISTOCRATS" OF PHYSIC.

It is believed by many impecunious persons, medical practitioners and others, that those gentlemen who hold the positions of lecturers and teachers at the London hospitals and medical schools have a royal time of it. The family doctor envies them their exalted professional rank; he looks upon them as fortune's favourites; he believes that every one of them secures a fat income from students' fees; and that, in addition, their private practice, as the result of their position at the hospital, produces a steady and continuous daily flow of guineas, which ceases not "from morn till dewy eve." If any hospital lecturer should happen to read this, he will probably feel a little more exasperated than usual when he makes comparison between the golden vision and the actual realities of the case. For though the hospital lecturer as a rule is by no means a professional Crœsus, he feels that he ought to be one. There are not many persons who are more inclined to magnify their office than hospital lecturers; and it cannot be denied that their office is really important, and that in not a few instances they themselves confer honour upon it, as much or more than they are honoured by it. The lecturer is always a man of some scientific attainments, and in a good many instances he really deserves the title of "man of science."

The Royal Commission on Hospitals has pricked several very pretty bubbles; and one of those which it has quite effectually burst is the delusion that hospital lecturers are "bloated aristocrats" in point of pay. Goldsmith's village parson of last century was "Passing rich with forty pounds a year." This is the very indentical sum which certain lecturers at the London hospitals can claim as their only reward. For this, certain of them deliver at least four lectures, each of an hour's duration, every week. That is to say, they receive somewhat less than 10s. a lecture. When we consider that a prima donna is not seldom paid £100 for a much shorter performance, we shall not need to be asked twice to spare a little sympathy for the London medical educator of his youthful fellow-countrymen. There is a certain grim humour in Section 519 of page 86 of the Report of the Lords' Commission, which states that "Evidence was given respecting the expenses of the (medical) schools, and the mode in which the professors and teachers are paid. Speaking generally, the remuneration of the teaching staff is certainly not high." It certainly is not "high," as a few facts culled from the Report will very convincingly show.

St. Thomas's is by no means a second-rate hospital, nor does it employ second-rate professors and teachers. But it was given in evidence before the Commission that the very largest amount paid to a medical professor at St. Thomas's is £240 a year; and this includes the remuneration for "practice" as well as for lectures. The happy recipient of the £240 a year is at the very topmost rung of the professional ladder. His "bloated" fellow-professor at the opposite end of the scale is proud to put £40 a year into his pocket. "Only that, and nothing more." But St. Thomas's, as everybody knows, is one of the richest of the London hospitals. At St. George's the senior professor receives no more than £100 a year. What may be the pay of the junior we hesitate even to speculate. But if it bears the same ratio to the senior's magnificent income as is borne at St. Thomas's, it cannot be much more than 5s. or 6s. a week, about £15 or £16 a year. It is to be hoped that this gentleman has learnt, like Succi, the secret of maintaining life on some rare and miraculous drug. It is certain, since he must have clothes to lecture in, that he cannot have anything to spare for food. The lecturers at St. Bartholomew's and at University and King's Colleges are paid a little better than this; but those at most of the other hospitals receive about the same rate of remuneration as has been given for St. Thomas's and St. George's.

If evolution has been the prominent characteristic in the development of any class of social institutions, it has surely been especially so in the case of hospitals. Every one of them has had a purely spontaneous origin. Their constitutions, their methods, their inconsistencies, and the odd results, some of which we have just been considering, are all characteristics of an evolutionary process rather than products of philosophical reason directed to intelligent ends. But reason, it must be admitted, sometimes produces more intelligent results than can be claimed for spontaneous evolution. It might have been better in many respects if hospitals could have had the benefit of a little more intelligence in their origin and later administration. But, however that may be, there is no doubt that the clear light thrown upon them recently by the Lords' Committee, has revealed certain not very pleasing anomalies, which an intelligence as practical as it is luminous, will make all possible haste to get rid of. The small fees paid to our medical lecturers are absurd. But if they are in any sense indicative of the actual value of the services rendered to medicine and the public by those lecturers, then we have an anomaly which is not only absurd but scandalous.

It is a proposition which generally holds good that a thing is worth what it will fetch in the market. There are, no doubt, exceptions to this rule, but the exceptions, it must be remembered, are exceptions. The rule still remains the rule. Will £40 a year command a teacher who is worth £400? As a rule it will not. Will a £100 a year command a professor who is worth a £1,000? Again the answer must be that it may sometimes, but the chances are all the other way. Yet, practically, all the London medical schools act as if they could rely upon securing scores of teachers, every one of whom will work for about a tenth part of the remuneration which he really ought to receive. This we venture to say is economics of the most dangerous kind. Human nature is such that ninety-nine men in every hundred who are paid £40 a year will do work worth £40 a year—that or less, rather than more. Now, a reform is needed here. Instead of ten medical schools in London we ought to have two, or possibly even only one. This is a question which cannot be entered upon at the close of an article. But if London medicine is to be metropolitan and imperial, and not provincial and ridiculous, it must use the intelligence of the present time, and supplement with methods of reason the manifest defects and anomalies which are the natural products of a spontaneous and undirected process of evolution. We shall return to this subject on future occasions. The reforms we demand are imperative, and must be effected.

